# Editorial: Between emotional regulation and dysregulation: perspectives, interventions, tools and technologies for psychological well-being

**DOI:** 10.3389/fnbeh.2026.1843064

**Published:** 2026-05-07

**Authors:** Luigia Simona Sica, Michela Ponticorvo, Alessandro Frolli

**Affiliations:** 1 Department of Humanities, University of Naples Federico II, Naples, Italy; 2 Università degli Studi Internazionali di Roma, Rome, Italy

**Keywords:** emotion dysregulation, emotion regulation, flexibility, mHealth, psychological interventions

The topic of emotional development, recognition, management, and regulation of emotions is a central theme in developmental psychology. The individual's ability to live in harmony with their emotional conditions is central to psychological wellbeing and psycho-social balance. On the contrary, difficulty in recognizing and managing one's emotions can become a factor of discomfort for the individual and psycho-social risk. This is true throughout life, and especially in those phases of transition (adolescence, emerging adulthood, and aging) in which the individual is immersed in a world of internal and external changes that requires the ability to redefine oneself and rebalance bio-psycho-social. It is for this reason that the regulation of emotions is in our opinion a central element for the development of positive life trajectories, for psychological wellbeing and for psycho-social adaptation. In this topic, through multidisciplinary contributions and different theoretical and methodological perspectives, we will address the issue of emotional regulation/dysregulation with the aim of deepening risk and protection factors, as well as tools and methods of investigation and intervention.

In this perspective, the set of scientific contributions collected in the current Topic approaches the complexity of the concept of emotional regulation, illustrating the elements of flexibility recently explored by the scientific literature. Contemporary research, indeed, conceptualizes emotion regulation not merely as the use of specific strategies, but as emotion regulation flexibility—the ability to contextually switch between strategies based on situational demands (Gross and Ford, 2024; Wang and Hawk, 2022b). Developmental models now emphasize that emotion regulation is a dynamic, within-person process, with studies showing, for instance, that large part of variance in adolescent regulation occurs at the daily, situational level rather than being a stable trait (De France and Hollenstein, 2022). In this context, the emergent concept of emotion regulation flexibility represents a shift in developmental and clinical psychology from identifying healthy vs. unhealthy strategies to evaluating the person-context fit of emotional management (Bonanno and Burton, 2013). In this way, it is defined as the ability to effectively synchronize one's regulatory efforts with the fluctuating demands of the environment (Aldao et al., 2015). In fact, by repositioning the conception of emotional regulation within a paradigm of complexity, the flexibility framework posits that any strategy can be functional depending on the situational context and the individual's goals. Indeed, according to developmental approach, emotion regulation flexibility is considered a critical protective factor against psychopathology, particularly during adolescence, when social environments become increasingly complex and demanding (Wang and Hawk, 2022a). It was also found to be associated with greater resilience and lower symptoms of anxiety and depression, whereas regulatory rigidity is a hallmark of emotional dysregulation.

This approach should also be considered when emotional dysregulation processes are observed. On the other hand, for some time now, the research found that dysregulation is often viewed not as a lack of regulation, but as the employment of strategies that are ineffective for the current context (Cole et al., 1994). Thus, emotion dysregulation is increasingly viewed through a transdiagnostic lens, acting as a core mechanism linking adverse childhood experiences (ACEs) to later psychopathology, including depression, anxiety, and ADHD (Bakken et al., 2025).

Finally, looking at today's society, the digital context cannot be overlooked. In this case, new digital emotion regulation models address how youth now regulate emotions through digital means (e.g., social media), distinguishing between digitally induced emotions and digitally regulated states (Hollenstein and Faulkner, 2024).

Therefore, overall, in the light of the existing empirical evidence, it is possible to consider modern emotion regulation development as a hybrid process involving biological maturation, individual skills, social co-regulation, and digital environmental affordances.

The contributions featured in this topic summarize the complexity of this definitional framework, and, in their multiplicity of angles and targets, contribute to exploring the relationship between emotional regulation and dysregulation from a perspective of life span psychology. Specifically, the topic identifies three core components of emotion regulation flexibility: protective and risk factors in a life span perspective (childhood and transition to adulthood; Aykut and Kara; Gavrilova and Veraksa; Cecere et al.; Yu and Liu; Minichiello et al.) and in old age (Cho and Choi); interventions with non-normative participants or clinical groups (Caldiroli et al.; Sebri et al.; Morozova et al.; Liu et al.; Xia et al.; Dagnino et al.; Konrad et al.; Secer et al.; Dragic); digital emotion regulation (Nguyen et al.) and new investigation tools (Gharamaleki and Fathipour-Azar; Lee et al.).
